# A new derivative for oxosteroid analysis by mass spectrometry

**DOI:** 10.1016/j.bbrc.2014.01.190

**Published:** 2014-04-11

**Authors:** K. Rigdova, Y. Wang, M. Ward, W.J. Griffiths

**Affiliations:** a College of Medicine, Swansea University, Singleton Park, Swansea SA2 8PP, UK; b Proteome Sciences plc, Coveham House, Downside Bridge Road, Cobham, Surrey KT11 3EP, UK

**Keywords:** API, atmospheric pressure ionization, CID, collision-induced dissociation, ESI, electrospray ionization, GC, gas chromatography, LC, liquid chromatography, MS, mass spectrometry or mass spectrum, MS/MS, tandem mass spectrometry, TMT, tandem mass tag, TMTH, tandem mass tag hydrazine, TOF, time-of-flight, SPE, solid phase extraction, Steroids, Tandem mass spectrometry, Derivatisation, Steroid glucuronides, Urine

## Abstract

Here we report a new method for oxosteroid identification utilizing “tandem mass tag hydrazine” (TMTH) carbonyl-reactive derivatisation reagent. TMTH is a reagent with a chargeable tertiary amino group attached through a linker to a carbonyl-reactive hydrazine group. Thirty oxosteroids were analysed after derivatisation with TMTH by electrospray ionization mass spectrometry (ESI-MS) and were found to give high ion-currents compared to underivatised molecules. ESI-tandem mass spectrometry (MS/MS) analysis of the derivatives yielded characteristic fragmentation patterns with specific mass reporter ions derived from the TMT group. A shotgun ESI-MS method incorporating TMTH derivatisation was applied to a urine sample.

## Highlights

• New derivatisation method for oxosteroids.
• Enhanced signal in ESI-MS and ESI-MS/MS.
• Informative MS/MS fragmentation.

## 1 Introduction

**Fig. 1:**
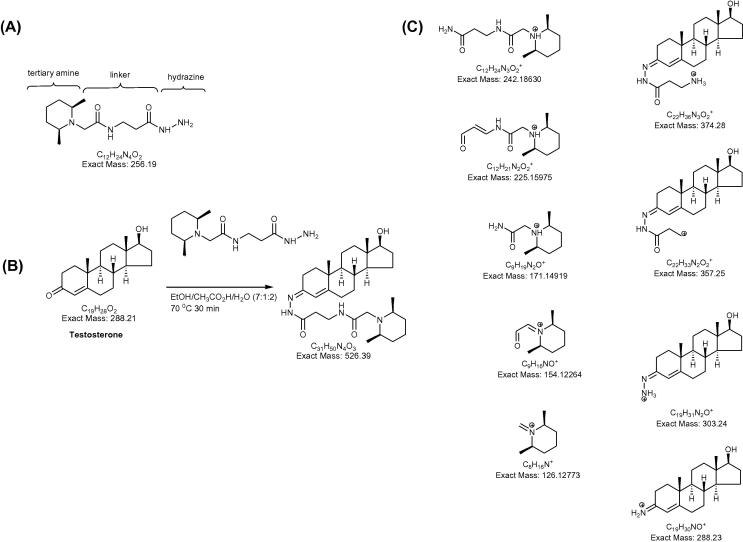
(A) Generic structure of the TMTH derivatisation reagent. (B) Derivatisation reaction of oxosteroids with TMTH exemplified by testosterone. (C) Structures of the major fragment ions observed in the MS/MS spectrum of the [M+H]^+^ ion of TMTH derivatised testosterone.

Steroid hormones are small molecules synthesized from cholesterol, mainly in cells of the adrenal cortex, the gonads, and the placenta. The initial step in steroid hormone biosynthesis is cleavage of the bond between C-20 and C-22 of the cholesterol side-chain in a reaction catalysed by the enzyme P450scc (CYP11A1) to give pregnenolone. This steroid is subsequently metabolised further by a number of enzymes to the classical steroid hormones. Steroid hormones have important biological functions regulating development and metabolism [1]. The analysis of steroid molecules by mass spectrometry (MS) presents a number of challenges: they are mostly involatile, thermally labile molecules and require derivatisation prior to gas-chromatography (GC)-MS analysis; many are hydrophobic and poorly soluble in aqueous solvents making liquid chromatography (LC) separations difficult; unconjugated steroids are neutral molecules and thus not optimal for atmospheric pressure ionisation (API)-MS; and regulatory steroids may be present in biological samples at very low levels [2]. To overcome some of these challenges we have adopted a strategy where oxosteroids are derivatised with the “tandem mass tag hydrazine” (TMTH) reagent making them suitable for subsequent API-MS analysis (Fig. 1). This strategy is applicable to the analysis of both free and intact steroid conjugates, such as steroid sulphates and glucuronides which require hydrolysis before GC–MS analysis.

Thomson et al. originally developed amine-reactive tandem mass tags (TMT) for labelling of peptides [3]. Here we apply carbonyl-reactive TMTH in the analysis of oxosteroids. The TMTH reagent (Fig. 1) consists of a carbonyl-reactive hydrazine group connected through a linker to a readily protonated tertiary amino group. TMTH has an exact mass 256.19 Da and introduces a mass shift to the derivatised molecule of 238.18 Da (Fig. 1).

The aim of this study was to develop an electrospray ionization (ESI)-MS “shotgun” method for analysis of oxosteroids using the TMTH derivatisation reagent. Thirty oxosteroids including androgens, endogenous and exogenous corticosteroids, estrogens and progestagens, were analysed following derivatisation by direct infusion ESI-MS and ESI-tandem mass spectrometry (MS/MS) using a quadrupole – time-of-flight (Q-TOF) instrument. The method has been applied to profile steroid metabolites in urine.

## 2 Methods

### 2.1 Reagents and standards

Steroid standards nandrolone (19-nortestosterone, N), testosterone (T), betamethasone (Bet), prednisolone (Pred), dexamethasone (Dex), flumethasone (Flu), beclomethasone (Bec), cortisol (hydrocortisone, F), cortisone (E), estrone (E1), 5α-dihydrotestosterone (DHT) and pregnenolone (3β-hydroxypregn-5-en-20-one) were from Sigma–Aldrich (Poole, Dorset, UK). Androstenedione (AD) was from Fluka (Germany). Tetrahydrodeoxycortisol (THS), tetrahydrodeoxycorticosterone (THDOC), tetrahydrocorticosterone (THB) and tetrahydrocortisol (THF), were from Steraloids Inc (Newport, RI, USA). 5α-Androsterone (A), estrone sodium sulphate (E1-S) and 17-hydroxypregnenolone (3β,17α-dihydroxypregn-5-en-20-one) were purchased from Makaira Ltd (London, England). Dehydroepiandrosterone (DHEA) was from Avanti Polar Lipids (Alabaster, AL, USA). Sodium DHEA-3-sulphate (DHEA-S) was from TCI (Tokyo, Japan). 5α-Androsterone glucuronide (A-GlcA) was from LGC Standards GmbH (Wesel, Germany). Other steroids, 3α-hydroxy-5β-pregnan-20-one, 3β-hydroxy-5β-pregnan-20-one, 3α-hydroxy-5α-pregnan-20-one, [11,11-^2^H_2_]-3β-hydroxy-5α-pregnan-20-one, 3β-hydroxypregn-4-en-20-one, 3β-hydroxypregn-5,16-dien-20-one, [3,4-^13^C_2_]-progesterone (pregn-4-ene-3,20-dione) were from previous studies [4]. For steroid structures see Supplementary Fig. S1.

Solid phase extraction (SPE) cartridges, certified Sep-Pak C18, 3cc, 200 mg were from Waters Inc. (Milford, Massachusetts, USA), Leur Lock syringes were from BD Biosciences, (Oxford, UK). Solvents were from Fisher-Scientific (Loughborough, Leicestershire, UK) of HPLC or Analytical grade. Acetic acid 100% AnalaR Normapur was from VWR International S.A.S, (Baire, France). The derivatisation reagent TMTH (Fig. 1) was supplied by Proteome Sciences plc. (Surrey, UK).

### 2.2 Derivatisation of oxosteroids with TMTH

1 mg of each steroid was dissolved in 1 mL ethanol and vortex mixed, then diluted with ethanol to give working solutions of 0.1 and 0.01 μg/μL. The derivatisation procedure was as follows: 5 mg of TMTH (0.02 mmole) was dissolved in 60 μL of ethanol. 10 μL of steroid working solution (about 3.5 or 0.35 nmole) was added, followed by 20 μL of water (HPLC grade) and 10 μL of glacial acetic acid. The mixture was vortex mixed and warmed at 70 °C for 30 min on block heater. The sample mixture was dried using Scanspeed vacuum concentrator (Coolsafe, West Sussex, UK) and reconstituted in 1 mL of 10% methanol.

A Sep-Pak C18 cartridge was attached to Agilent Manifold Processing Station (Agilent Technologies, Inc., Wilmington, USA) and vacuum applied. The sorbent was washed with 6 mL 100% methanol followed by an equilibration rinse with 6 mL of 10% methanol. Derivatised steroids reconstituted in 1 mL of 10% methanol were applied to the column. The column was washed with 6 mL of 10% methanol (to remove excess of derivatisation reagent) and steroid-hydrazones eluted using 3× or 4 × 1 mL of 100% methanol (depending on the steroid), producing fractions 1–4. Mono-derivatives were present predominantly in the first and second fractions. Double derivatives were present also in the third and fourth fractions.

### 2.3 ESI-MS and -MS/MS analysis of derivatised oxosteroids

ESI-MS and ESI-MS/MS analysis was performed on a Q-TOF (Waters, UK) using nano-ESI needles (EconoTips12, Presearch Ltd.). The ESI-MS and MS/MS conditions were as follows: spray voltage, 1.8 kV; collision gas, argon; collision voltage from 5 V for MS, 30–40 V for MS/MS; LM from 10 to 15; HM from 10 to 15; ion polarity, positive. The Q-TOF was calibrated externally by MS/MS using a [Glu^1^] Fibrinopeptide solution (1 pmol/μL in 60% acetonitrile, 0.1% formic acid) prior to each analytical session.

### 2.4 Preparation and analysis of urine

An SPE cartridge (200 mg Sep-Pak tC18), was washed with 4 mL of ethanol, followed by 4 mL of methanol and conditioned with 4 mL of HPLC water. 100 μL of urine was diluted with 1 mL of HPLC water and applied to the column. After washing with 3 mL of HPLC water, steroids were eluted with 4 mL of methanol and dried using a scanspeed vacuum concentrator. Before the drying was complete, 50 μL of underivatised steroid extract was subtracted for direct ESI-MS analysis of the underivatised steroids by negative ion-ESI. The fully dried extract was then derivatised with 5 mg of TMTH dissolved in 70 μL of ethanol, 20 μL water (HPLC grade) and 10 μL glacial acetic acid as described above.

## 3 Results

### 3.1 Amount of TMTH reagent required for effective derivatisation

In our initial protocol 5 mg of TMTH (0.02 mmole) was used to quantitatively derivatise the steroid standards (i.e. 0.1–1 μg, 0.35–3.5 nmole for testosterone) and there was no evidence for any unreacted steroids in the resultant ESI-MS spectra recorded. We thus investigated if the TMTH/steroid ratio of >5000:1 (mole ratio) could be reduced while still maintaining high derivatisation yields. In an initial experiment with testosterone and nandrolone at 10 μg of each steroid, 20 μg in total (70 nmole), and the TMTH concentration reduced to 0.1 mg (0.4 μmole TMTH, TMTH/steroid mole ratio 5.7:1), the yield of derivatised steroids was found to be the same as when 5 mg TMTH was used. When 1 μg of both nandrolone and of testosterone (2 μg total, 7 nmole) was used and the amount of TMTH reduced to 0.005 mg (20 nmole, TMTH/steroid mole ratio 2.8:1) the yield of derivatised steroid was the same as when 5 mg of TMTH was used indicating that a TMTH/steroid mole ratio of about 3:1 is sufficient for quantitative derivatisation in 100 μL of solvent.

### 3.2 ESI-MS analysis

**Fig. 2:**
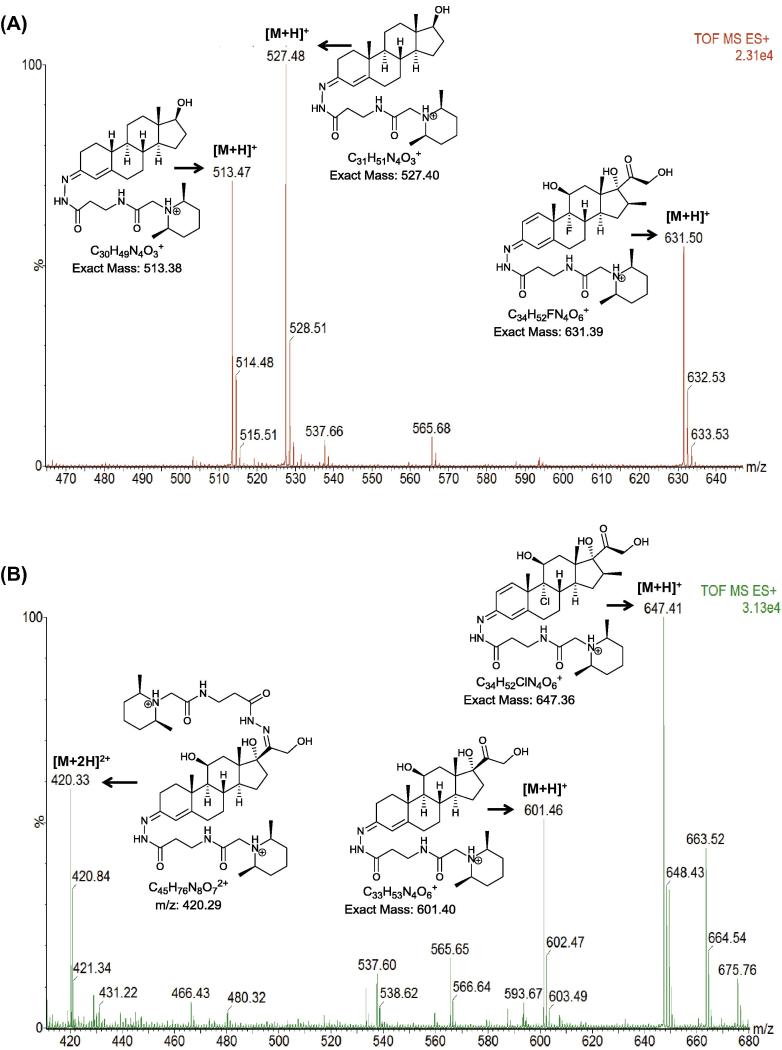
ESI-MS spectra of TMTH derivatised (A) nandrolone [M+H]^+^*m*/*z* 513, testosterone [M+H]^+^*m*/*z* 527 and betamethasone [M+H]^+^*m*/*z* 631, and (B) cortisol (double derivative) [M+2H]^2+^*m*/*z* 420, (single derivative) [M+H]^+^*m*/*z* 601, and beclomethasone (single derivative) [M+H]^+^*m*/*z* 647.

The derivatisation protocol was tested on thirty oxosteroids (Supplementary Fig. S1). Mono TMTH derivatives of oxosteroids give an [M+H]^+^ ion upon ESI (Fig. 2). When steroids with multiple oxo groups were analysed, single and/or double TMTH derivatives were observed (Fig. 2). The abundances of doubly derivatised steroids varied, depending on structure of the parent steroid. Most steroids with carbonyl groups at C-3 and C-17, or C-3 and C-20 give a mixture of single and double derivatives (Table S1), but in the case of betamethasone and beclomethasone double derivatives were not abundant despite the parent structure having carbonyl groups at C-3 and C-20. This is due to steric hindrance provided by the β-methyl group on C-16, not allowing sufficient space for a second TMTH tag to be attached to the steroid. The limit of detection of TMTH derivatised steroids was determined at a signal to noise ratio of 3:1 to be in the range of 2.5–5 pg/μL. Improvement in sensitivity, compared to underivatised steroid standards, upon ESI analysis in the positive mode ranged from 14-fold for androstenedione to 1457-fold for dehydroepiandrosterone and up to 2755-fold for dehydroepiandrosterone sulphate.

### 3.3 ESI-MS/MS analysis

**Fig. 3:**
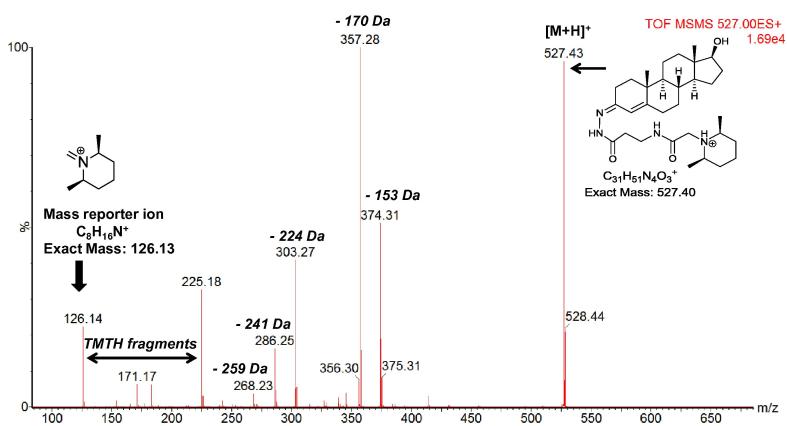
ESI-MS/MS spectrum of the [M+H]^+^ ion of TMTH derivatised testosterone *m*/*z* 527.

ESI-MS/MS was used to investigate the fragmentation profile of TMTH derivatised steroids. The parent [M+H]^+^ ion was submitted to collision-induced dissociation (CID) with argon gas at a collision energy of 30–40 eV, resulting in characteristic fragmentation patterns (Fig. 3). The dominant fragments ions present in the MS/MS spectra of derivatised steroids consist of the steroid skeleton with remnants of TMTH attached, and in the low *m*/*z* range fragment-ions of the TMTH derivatising group (Fig. 1). In the following the MS/MS spectra of the different structural classes of oxosteroids are described.

#### 3.3.1 MS/MS of androgens derivatised with TMTH (Table S2)

##### 3.3.1.1 3-Oxo-4-ene (nandrolone, testosterone) and 3-oxo (dihydrotestosterone) steroids

The dominant fragment ions are formed by loss of 153 (C_9_H_15_NO), 170 (C_9_H_18_N_2_O) and 224 Da (C_12_H_20_N_2_O_2_). Weaker fragments are formed from loss of 241 Da (C_12_H_23_N_3_O_2_) and of 259 Da (C_12_H_25_N_3_O_3_). Fragment ions derived from the TMTH tag are observed at *m*/*z* 126 (C_8_H_16_N)^+^, 171 (C_9_H_19_N_2_O)^+^, 183 (C_10_H_19_N_2_O^+^) and 225 (C_12_H_21_N_2_O_2_)^+^ (Table S2, Fig. 1).

##### 3.3.1.2 17-Oxosteroids (androsterone, dehydroepiandrosterone, DHEA, and DHEA-sulphate)

As with the 3-oxosterods the MS/MS spectrum of androsterone is dominated by the neutral losses of 153, 170 and 224 Da, with weaker fragment ions formed by neutral loss of 256 (C_12_H_24_N_4_O_2_) and 274 Da (C_12_H_26_N_4_O_3_). These latter two neutral-losses are characteristic TMTH derivatised 17-oxo steroids and can be used to differentiate between derivatives at the 17-oxo and 3-oxo groups. DHEA gives an essentially similar fragmentation pattern to androsterone, but the steroid ring containing fragment ions are 2 Da lighter as a consequence of the unsaturated B ring in DHEA. The major fragment ions of DHEA-sulphate result from the loss of the 80 (SO_3_) and 98 (H_2_SO_4_) Da from the sulphuric acid conjugating group. Subsequent fragmentation of the [M+H-98]^+^ ion gives ions characteristic of a 17-oxosteroid. Androsterone glucuronide shows the classical neutral loss of 176 Da for a steroid glucuronide giving the [M+H-176]^+^ ion as the major fragment ion.

The fragmentation pattern of androstenedione is very similar to that of both testosterone (3-oxo) and DHEA (17-oxo), the fragment ions at *m*/*z* 251 (−274 Da) and 269 (−256 Da) indicate that the 17-oxo group is derivatised to some extent.

#### 3.3.2 MS/MS of progestagens derivatised with TMTH (Table S3)

C_21_ progestagens showed consistent fragmentation patterns with abundant neutral losses similar to those observed in the androgens (−153, −170, −224, −241 Da) with additional, but less abundant, neutral losses of 113, 188, and 300 Da. Fragment ions derived from TMTH at *m*/*z* of 126, 171, 183, and 225 were observed as in the MS/MS spectra similar to the androgens.

#### 3.3.3 MS/MS of endogenous corticosteroids derivatised with TMTH

##### 3.3.3.1 Tetrahydrocorticosteroids (THDOC, THS, THB, THF, Table S4)

These four compounds are each derivatised at C-20 and all give a fragment ion at *m*/*z* 242 (C_12_H_24_N_3_O_2_)^+^ as the dominant peak in the MS/MS spectrum with other TMTH fragment ions being observed at *m*/*z* 225, 183, 171 and 126 (Table S4). In addition to the above fragments THDOC shows fragment ions at *m*/*z* 385 and 367 resulting from the neutral losses of 188 Da (C_9_H_18_N_2_O + H_2_O) and 206 Da (C_9_H_18_N_2_O + 2 × H_2_O) and at *m*/*z* 314 formed by the neutral loss of 259 Da (C_12_H_23_N_3_O_2_ + H_2_O). The isomers THS and THB give similar MS/MS spectra, but they are not identical. Both loose water more readily than THDOC and this is reflected by the presence of multiple fragment ions derived from the neutral loss of portions of the TMTH group accompanied by loss of water i.e. −171 Da (C_9_H_15_NO + H_2_O), −188 (C_9_H_18_N_2_O + H_2_O), −206 (C_9_H_18_N_2_O + 2 × H_2_O), −242 Da (C_12_H_20_N_2_O_2_ + H_2_O). The isomers can be differentiated by the enhanced abundance of peaks at *m*/*z* 365 (−224 Da either loss of C_12_H_20_N_2_O_2_ or C_9_H_18_N_2_O − 3 × H_2_O) and 300 in the spectrum of THB, while peaks at *m*/*z* 302 and 384 are more abundant in the spectrum of THS.

THF has one extra hydroxyl group compared to THS and THB. The MS/MS spectrum of THF shows loss of one, two and three water molecules. As was the case with THS and THB multiple fragment ions derived from the neutral loss of portions of the TMTH group accompanied by loss of water are observed i.e. −189 (C_9_H_15_NO + 2 × H_2_O), −206 (C_9_H_18_N_2_O + 2 × H_2_O), −224 Da (either loss of C_12_H_20_N_2_O_2_ or of C_9_H_18_N_2_O + 3 × H_2_O) and −242 Da (loss of C_12_H_20_N_2_O_2_ + H_2_O or of C_9_H_18_N_2_O + 4 × H_2_O). A major pair of fragment ions are observed at *m*/*z* 271 and 253. These probably correspond the ABCD ring structure with one hydroxyl and two double bonds and to the ring structure with three double bonds, respectively. Analogous fragment ions are observed in the spectrum of THS at *m*/*z* 273 and 255 where there is one less double bond in the ring system.

##### 3.3.3.2 Cortisol (F) and cortisone (E, Table S5)

The presence of more than one oxo group on these molecules results in the formation of both mono- and *bis* derivatives. Considering the mono-derivatives, the MS/MS spectra are more compatible with the derivative being at the C-3 position (cf. testosterone) than at the C-20 position (e.g. THDOC) in that the dominant fragment ions are due to the neutral losses of 153, 170, 224 and 241 Da derived from the loss of portions of TMTH as neutral fragments opposed to being fragment-ions of TMTH itself (Table S5).

### 3.4 Analysis of TMTH derivatised steroids in human urine

**Fig. 4:**
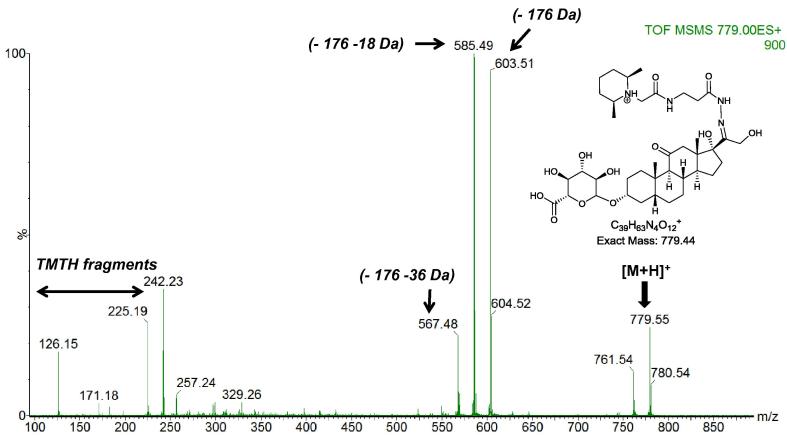
ESI-MS/MS spectrum of the [M+H]^+^ ion at *m*/*z* 779 in a human urine sample assigned to TMTH derivatised tetrahydrocortisone glucuronide.

In this preliminary study we used the shotgun ESI-MS and ESI-MS/MS method for evaluating abundances of steroid metabolites in human urine. TOF-MS and MS/MS analysis of human urine samples revealed abundant peaks corresponding to protonated TMTH derivatised glucuronides. The identifications were based on exact mass and fragmentation patterns. The major steroids were characterised as glucuronides by the neutral loss of 176 Da (Fig. 4). By fragmenting the [M+H-176]^+^ ions generated in the ESI source in a pseudo MS/MS/MS experiment and comparing the resulting spectra to those of the unconjugated standards it was possible to identify the steroids present to be glucuronides of androsterone and/or etiocholanolone, hydroxylated androsterone and or hydroxylated etiocholanolone, tetrahydrodeoxycortisol and/or tetrahydrocorticosterone, tetrahydrocortisone and tetrahydrocortisol and/or cortolone.

In conclusion, we present a simple, mild and fast derivatisation method for oxosteroid analysis by shotgun ESI-MS and MS/MS. We applied the method to thirty oxosteroids, whose ionization efficiencies increased from 14 up to 2755-fold when compared to their underivatised equivalents. TMTH tags were tested on urine samples, showing that direct analysis of steroid conjugates is possible.

## Appendix A Supplementary data

Supplementary data 1 Structures of steroid compound investigated in the current study.
