# Supplementary material for: A new derivative for oxosteroid analysis by mass spectrometry
Source: Biochem Biophys Res Commun. 2014 Apr 11;446(3):762–7. doi: 10.1016/j.bbrc.2014.01.190 (PMC4000438; doi:10.1016/j.bbrc.2014.01.190)
Supplement: Supplementary data 1 — Structures of steroid compound investigated in the current study. [file mmc1.ppt]

## Slide 1
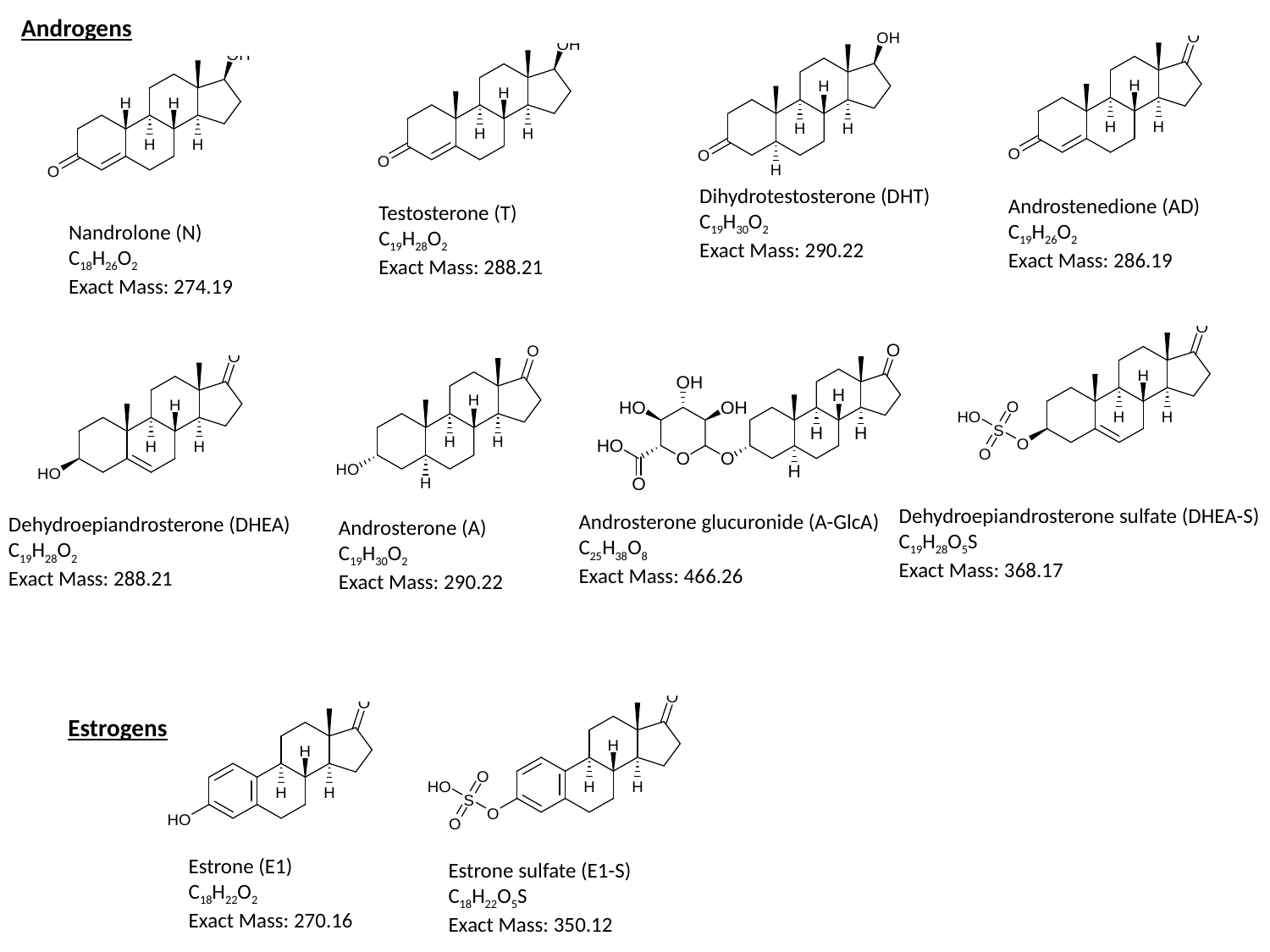

Androgens
Dihydrotestosterone (DHT)
C19H30O2
Exact Mass: 290.22
Androstenedione (AD)
C19H26O2
Exact Mass: 286.19
Testosterone (T)
C19H28O2
Exact Mass: 288.21
Nandrolone (N)
C18H26O2
Exact Mass: 274.19
Dehydroepiandrosterone sulfate (DHEA-S)
C19H28O5S
Exact Mass: 368.17
Androsterone glucuronide (A-GlcA)
C25H38O8
Exact Mass: 466.26
Dehydroepiandrosterone (DHEA)
C19H28O2
Exact Mass: 288.21
Androsterone (A)
C19H30O2
Exact Mass: 290.22
Estrogens
Estrone (E1)
C18H22O2
Exact Mass: 270.16
Estrone sulfate (E1-S)
C18H22O5S
Exact Mass: 350.12

## Slide 2
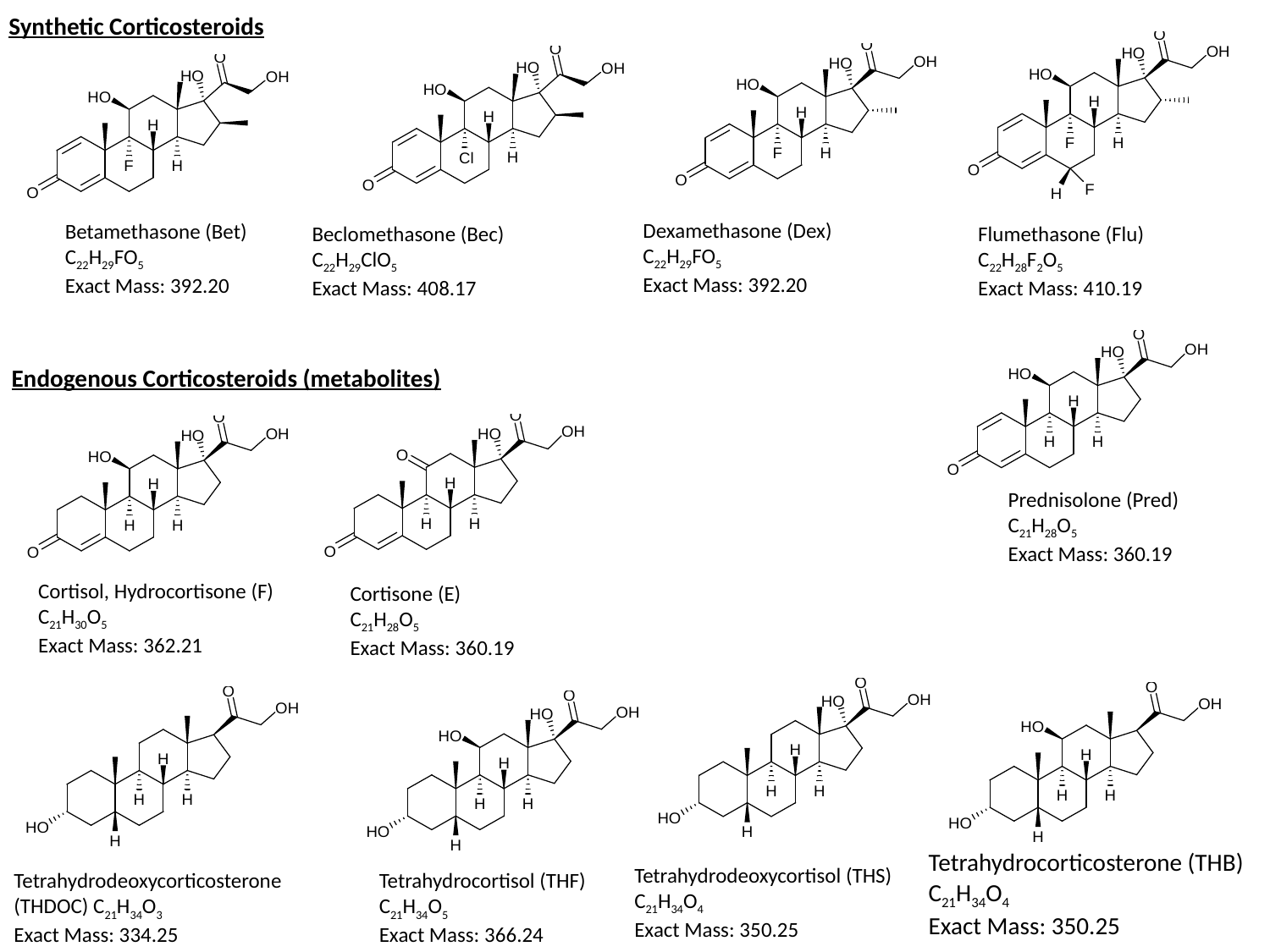

Synthetic Corticosteroids
Dexamethasone (Dex)
C22H29FO5
Exact Mass: 392.20
Betamethasone (Bet)
C22H29FO5
Exact Mass: 392.20
Beclomethasone (Bec)
C22H29ClO5
Exact Mass: 408.17
Flumethasone (Flu)
C22H28F2O5
Exact Mass: 410.19
Endogenous Corticosteroids (metabolites)
Prednisolone (Pred)
C21H28O5
Exact Mass: 360.19
Cortisol, Hydrocortisone (F)
C21H30O5
Exact Mass: 362.21
Cortisone (E)
C21H28O5
Exact Mass: 360.19
Tetrahydrocorticosterone (THB)
C21H34O4
Exact Mass: 350.25
Tetrahydrodeoxycortisol (THS)
C21H34O4
Exact Mass: 350.25
Tetrahydrodeoxycorticosterone
(THDOC) C21H34O3
Exact Mass: 334.25
Tetrahydrocortisol (THF)
C21H34O5
Exact Mass: 366.24

## Slide 3
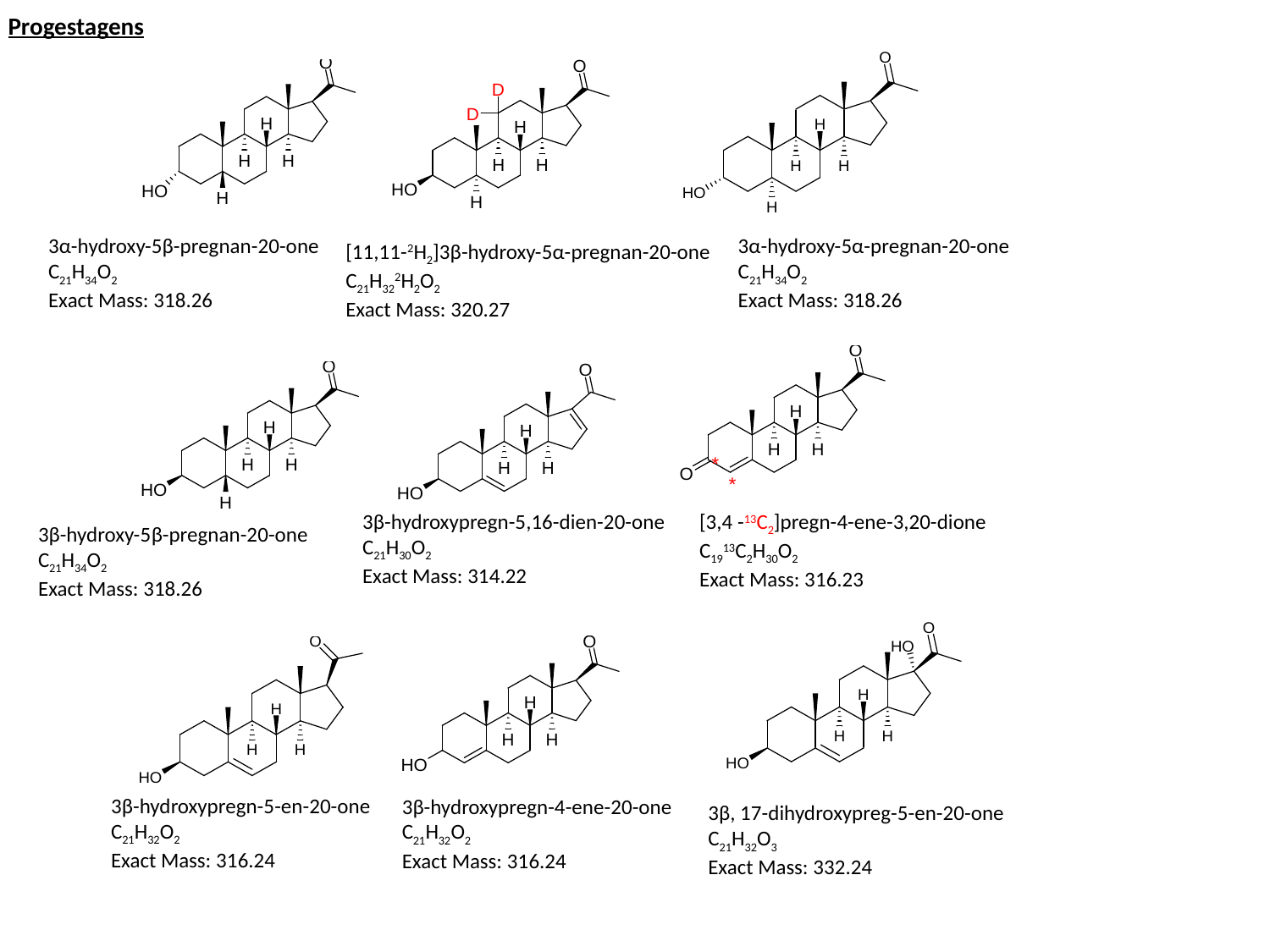

Progestagens
3α-hydroxy-5α-pregnan-20-one
C21H34O2
Exact Mass: 318.26
3α-hydroxy-5β-pregnan-20-one
C21H34O2
Exact Mass: 318.26
[11,11-2H2]3β-hydroxy-5α-pregnan-20-one
C21H322H2O2
Exact Mass: 320.27
3β-hydroxypregn-5,16-dien-20-one
C21H30O2
Exact Mass: 314.22
[3,4 -13C2]pregn-4-ene-3,20-dione
C1913C2H30O2
Exact Mass: 316.23
3β-hydroxy-5β-pregnan-20-one
C21H34O2
Exact Mass: 318.26
3β-hydroxypregn-5-en-20-one
C21H32O2
Exact Mass: 316.24
3β-hydroxypregn-4-ene-20-one
C21H32O2
Exact Mass: 316.24
3β, 17-dihydroxypreg-5-en-20-one
C21H32O3
Exact Mass: 332.24
