# Supplementary material for: A new derivative for oxosteroid analysis by mass spectrometry
Source: Biochem Biophys Res Commun. 2014 Apr 11;446(3):762–7. doi: 10.1016/j.bbrc.2014.01.190 (PMC4000438; doi:10.1016/j.bbrc.2014.01.190)
Supplement: Supplementary data 2 — Supplementary Tables. [file mmc2.docx]

Table S1. Derivatisation state of steroids with two or more oxo groups

| Steroid | mono-derivative | double derivative |
| --- | --- | --- |
| *Methyl group not present on C-16* |  |  |
| Prednisolone | + | + |
| Cortisol | + | + |
| Cortisone | + | + |
| *α-Methyl group present on C-16* |  |  |
| Dexamethasone | + | + |
| Flumethasone | + | + |
| *β-Methyl group present on C-16* |  |  |
| Betamethasone | + | - |
| Beclomethasone | + | - |

Table S2. Major neutral loss and fragment ions observed in the MS/MS spectra of TMTH derivatised androgens

|  | % Relative Abundance | | | | | | | |
| --- | --- | --- | --- | --- | --- | --- | --- | --- |
| [M+H]^+^ | Neutral loss (Da) | | | | Fragment ion (*m/z*) | | | |
|  | -153 | -170 | -224 | -241 | 225 | 183 | 171 | 126 |
| 3-Oxo-4-ene |  |  |  |  |  |  |  |  |
| Nandrolone | 35 | 100 | 35 | 15 | 20 | 5 | 5 | 15 |
| Testosterone | 50 | 100 | 40 | 15 | 35 | 5 | 5 | 20 |
| 3-Oxo |  |  |  |  |  |  |  |  |
| Dihydrotestosterone | 55 | 100 | 35 | 15 | 75 | 20 | 15 | 70 |
| 17-Oxo |  |  |  |  |  |  |  |  |
| Androsterone | 55 | 45 | 30 | 15 | 100 | 15 | 15 | 80 |
| Dehydroepiandrosterone | 50 | 60 | 35 | 15 | 100 | 20 | 15 | 75 |
| 3,17-Dione |  |  |  |  |  |  |  |  |
| Androstenedione | 50 | 100 | 40 | 25 | 80 | 20 | 15 | 75 |
| DHEA-3-sulphate |  |  |  |  |  |  |  |  |
| [M+H-80]^+^ | 10 | 15 | 5 | 5 | 100 | 10 | 15 | 75 |
| [M+H-98]^+^ | 50 | 35 | 15 | 15 | 100 | 10 | 15 | 75 |

Table S3. Major neutral loss and fragment ions observed in the MS/MS spectra of TMTH derivatised progestagens.

|  | % Relative Abundance | | | | | | | | | | | | | |
| --- | --- | --- | --- | --- | --- | --- | --- | --- | --- | --- | --- | --- | --- | --- |
| [M+H]^+^ | Neutral loss (Da) | | | | | | | | | Fragment ion (*m/z*) | | | | |
|  | -113 | -153 | -170 | -188 | -224 | -241 | -255 | -258 | -300 | 242 | 225 | 183 | 171 | 126 |
| 3α-Hydroxy-5β-pregnan-20-one | 5 | 85 | 55 | 15 | 40 | 35 | 10 | 5 | 5 | 20 | 100 | 15 | 20 | 90 |
| 3β-Hydroxy-5β-pregnan-20-one | 5 | 75 | 45 | 10 | 30 | 25 | 5 | 5 | 5 | 15 | 95 | 15 | 20 | 100 |
| 3α-Hydroxy-5α-pregnan-20-one | 5 | 90 | 60 | 10 | 40 | 35 | 10 | 10 | 10 | 20 | 100 | 15 | 20 | 90 |
| [11,11-^2^H_2_]3β-Hydroxy-5α-pregnan-20-one | 5 | 85 | 65 | 5 | 45 | 35 | 15 | 10 | 5 | 20 | 95 | 20 | 20 | 100 |
| 3β-Hydroxypregn-5-en-20-one | 5 | 70 | 60 | 5 | 40 | 30 | 10 | 5 | 5 | 15 | 95 | 20 | 20 | 100 |
| 3β-Hydroxypregn-5-en-20-one | 5 | 70 | 55 | 5 | 35 | 30 | 10 | 5 | 5 | 10 | 90 | 15 | 20 | 100 |
| 3β-Hydroxypregn-5,16-dien-20-one | 5 | 65 | 100 | 5 | 55 | 35 | 5 | 10 | 5 |  | 75 | 15 | 15 | 65 |
| [3,4-^13^C_2_]Progesterone | 5 | 55 | 100 | 5 | 45 | 20 |  |  | 5 | 5 | 35 | 5 | 5 | 30 |

Table 4SA. Major neutral loss and fragment ions observed in the MS/MS spectra of TMTH derivatised tetrahydrocorticosteroids.

|  | % Relative Abundance | | | | | | | | |
| --- | --- | --- | --- | --- | --- | --- | --- | --- | --- |
|  | Neutral loss (Da) | | | Fragment ion (*m/z*) | | | | | |
| [M+H]^+^ | -18 | -36 | -206 | 256 | 242 | 225 | 183 | 171 | 126 |
| Tetrahydrodeoxy-  corticosterone (THDOC) | 20 | 5 | 10 | 10 | 100 | 70 | 15 | 10 | 70 |
| Tetrahydrodeoxycortisol (THS) | 90 | 10 | 10 | 10 | 100 | 65 | 15 | 10 | 55 |
| Tetrahydrocorticosterone (THB) | 60 | 15 | 10 | 10 | 100 | 65 | 15 | 10 | 65 |
| Tetrahydrocortisol (THF) | 50 | 50 | 5 | 5 | 100 | 55 | 10 | 10 | 45 |

Table S5. Major neutral loss and fragment ions observed in the MS/MS spectra of TMTH derivatised corticosteroids.

|  | % Relative Abundance | | | | | | | | | | | | | | | | | |
| --- | --- | --- | --- | --- | --- | --- | --- | --- | --- | --- | --- | --- | --- | --- | --- | --- | --- | --- |
| [M+H]^+^ | Neutral loss (Da) | | | | | | | | Fragment ion (*m/z*) | | | | | | | | | |
|  | -153 | -170 | -182 | -200 | -224 | -230 | -241 | -254 | 342 | 341 | 324 | 323 | 313 | 295 | 225 | 183 | 171 | 126 |
| Cortisone | 25 | 100 | 5 | 10 | 50 | 5 | 15 | 5 | 5 | 5 | 10 | 10 | 5 | 5 | 25 | 5 | 5 | 20 |
| Cortisol | 45 | 100 | 5 | 10 | 55 | 5 | 15 | 5 | 5 | 5 | 5 | 5 | 5 | 5 | 55 | 10 | 10 | 50 |
